# An Easy Way to Show Memory Color Effects

**DOI:** 10.1177/2041669516663751

**Published:** 2016-08-01

**Authors:** Christoph Witzel

**Affiliations:** Laboratoire Psychologie de la Perception, Université Paris Descartes, Paris, France

**Keywords:** cognition, color, experience/learning/expertise, memory, memory colors, objects and features, perception

## Abstract

This study proposes and evaluates a simple stimulus display that allows one to measure memory color effects (the effect of object knowledge and memory on color perception). The proposed approach is fast and easy and does not require running an extensive experiment. It shows that memory color effects are robust to minor variations due to a lack of color calibration.

## Introduction

A seminal series of studies showed that memory modulates color appearance (Hansen, Olkkonen, Walter, & Gegenfurtner, 2006; Olkkonen, Hansen, & Gegenfurtner, 2008). They used an achromatic adjustment procedure to show that observers see images of fruits as colored in their typical hues when their physical color actually corresponds to the neutral gray of the background. Since then, this method has been successfully employed to provide further evidence for memory color effects (Witzel, Valkova, Hansen, & Gegenfurtner, 2011; Kimura et al., 2013).

However, the achromatic adjustment method is rather complicated: It involves a color adjustment technique, with online computations that translate the observers’ key presses into polar transformations of colors on a calibrated computer display. The resulting effects are subtle and require running an extensive experiment to reveal them. Under some circumstances, it may be necessary to determine memory color effects in a simpler and faster way. This can be the case, for example, when comparing memory color effects across experimental conditions, across groups of observers, or across large samples of different stimuli. Moreover, it has been argued that many alleged top-down effects on perception are merely judgement or response biases and could not be observed in a display that shows images side by side, allowing for direct perceptual comparison (Firestone & Scholl, 2015).

**Figure 1.**
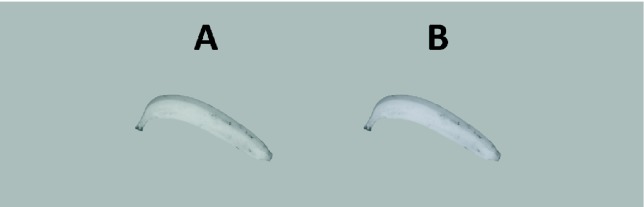
Main stimulus display. The left banana is completely gray (like the background), the right one slightly bluish. Which banana looks gray? If neither looks gray choose the most gray one.

**Figure 2.**
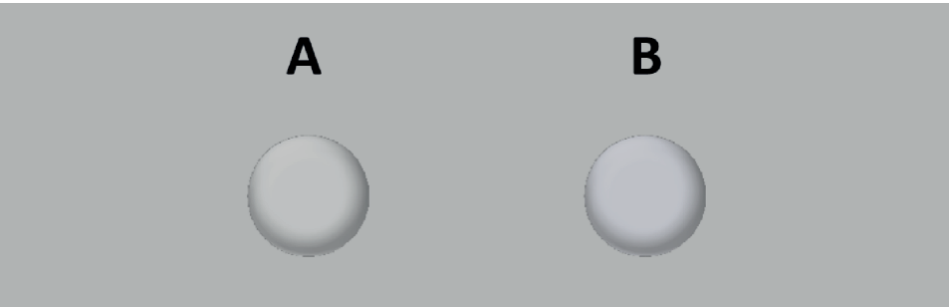
Control stimuli. Chromatic properties as for stimuli in Figure 1.

This claim can be quickly and easily tested with the stimuli in Figures 1 and 2: The disk and the banana on the left side of the figure have the same achromatic color (gray) as the background. The objects on the right side are more bluish than the gray background. The difference between the objects on the left and right corresponds to the color of the banana that observers adjusted in the seminal study of Hansen et al. (2006): On average, observers needed that much bluish tint to consider the banana as completely gray (Witzel, Olkkonen, & Gegenfurtner, in press).

These images in Figures 1 and 2 were produced assuming the characteristics of a typical computer monitor. Their precise chromatic properties may vary across different computer displays. However, the left stimulus stays at the chromaticity of the background, and the right stimulus remains more bluish across different displays. If memory color effects are robust, they might hold against minor variations in chromaticity.

## Study 1

A series of online surveys has been conducted to test this idea. In these surveys, the images were shown side by side, and observers were asked to indicate which one is most purely gray. In this way, observers were elicited to directly compare the completely gray and the bluish objects on perceptual grounds. The comparison of the two disks allows for assessing the proportion of choices when objects do not have a typical color and hence memory cannot influence perception. The comparison of the two bananas allows for assessing a memory color effect: Observers should choose the blue banana over the gray one at a proportion that is higher than chance. This should also be the case when accounting for the disk choices: The blue banana should be chosen more often than the blue disk. We conducted a large online survey, in which we presented the pair of disks and the pair of bananas at different pages of the survey. The pair of disks and the pair of bananas could not be seen simultaneously and could not be compared. Observers were asked to decide whether A or B looked most gray. Overall, 354 (age: 36.8 ± 12.1 years; 256 women) voluntary observers participated (for details see Methods section).

**Figure 3.**
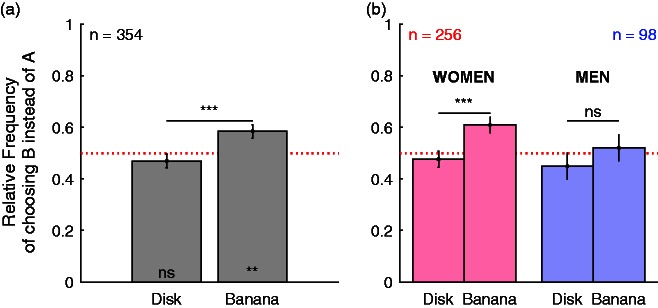
Results of Study 1. Panel a illustrates results for all participants together, Panel b separately for women (pink bars) and men (blue bars). The *y* axis represents the relative frequency of choosing the bluish over the gray exemplar. Error bars show standard errors of mean, the dotted red line chance probability. Symbols at the bottom of the bars indicate significance in binomial tests for differences from chance probability. Symbols on top of the horizontal bars indicate two-tailed significance in McNemar’s exact tests of differences between disk and banana. *** *p* < .001; ** *p* < .01; “ns” nonsignificant.

Figure 3(a) shows the relative frequency of choosing the more bluish over the gray exemplar of the respective object. A two-tailed binomial test was used to test whether relative frequencies of choosing one over the other alternative (A vs. B) differed from chance (*p* = .5). The first bar of Figure 3(a) shows the results for the two disks. Observers had a tendency to choose the gray disk more often (*k* = 188) than the bluish one (*n–k* = 166), but this tendency was not significant (*p* = .26). The fact that observers do not always choose the gray over the blue disk is in line with the blue bias in gray perception (Pearce, Crichton, Mackiewicz, Finlayson, & Hurlbert, 2014; Winkler, Spillmann, Werner, & Webster, 2015).

In contrast, observers chose the bluish banana (*n–k* = 207) significantly more often (*p* = .002) than the purely gray one (*k* = 147). This is illustrated by the second bar in Figure 3(a). Most importantly, the frequency of choosing the blue exemplar over the gray one was significantly higher for the pair of bananas than for the pair of disks, as shown through a two-tailed McNemar’s exact test (*p* < .00001). The Odds ratio of this difference is 3.1 and phi is 0.24, indicating a weak effect size (phi below 0.3 following Cohen’s, 1988 recommendations). The comparatively small effect size can be explained by the lack of display calibration. However, the advantage of online surveys is that large samples of participants can be easily measured. Averaging across large samples may cancel the noise and hence compensate for the low effect size. The fact that this online survey provides evidence for a memory color effect despite noise, and low effect size is most probably due to the large sample of participants (*n* = 354).

The present data can be used to compare different groups of observers. To illustrate this idea, memory color effects were compared between women and men. The comparison between women and men has no theoretical motivation and is done to illustrate the potential use of this method. Figure 3(b) shows the results separately for women (pink bars) and men (blue bars). In line with a memory color effect, McNemar’s exact test showed that women chose much more often the blue over the gray banana than they chose the blue over the gray disk (*p* = .00003; Odds ratio = 3.4; phi = 0.26). However, for men, this test was not significant (*p* = .34; Odds ratio = 2.2, phi = 0.14). To compare the strength of the memory color effect between women and men, the McNemar test has been extended by a *z* test that compares the proportion of blue banana choices relative to blue disk choices between women and men. Details are provided in the Appendix. The difference between women and men was not significant (*z* = −.76, *p* = .45). Hence, the absence of a memory color effect for men might well be due to the fact that the participant sample included many fewer men (*n* = 98) than women (*n* = 256).

## Study 2

**Figure 4.**
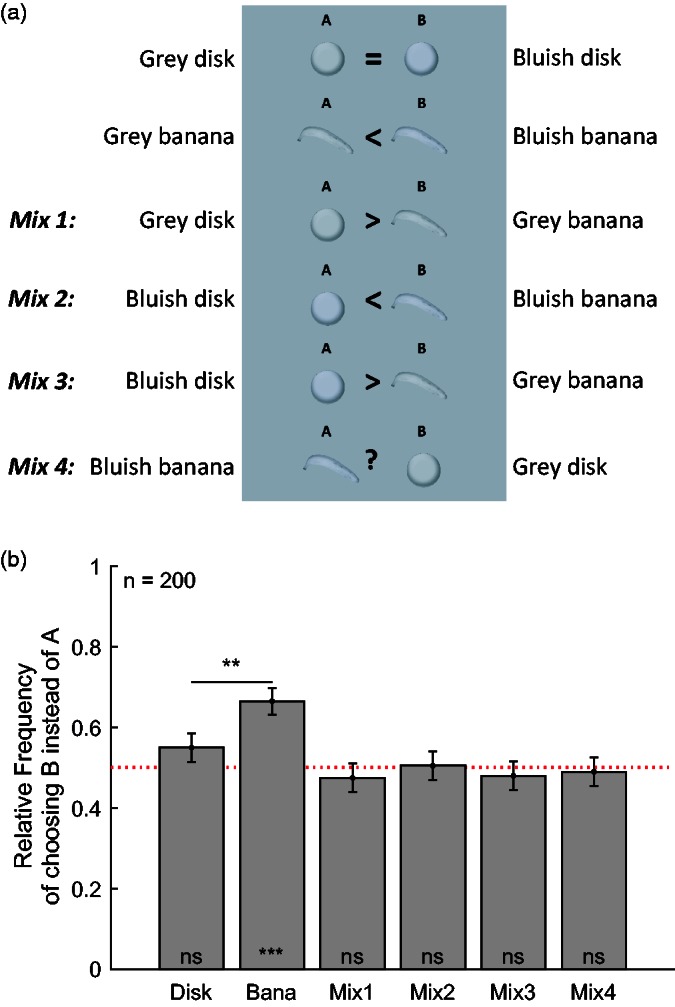
Stimuli and results of Study 2. The rows in Panel a show the six stimulus pairs in the order of appearance across the different pages of the survey. The symbols between the two objects of each pair were not part of the stimulus display but have been added to indicate the predictions based on the memory color effect. Panel b shows the relative frequency of choosing answer B over A for each stimulus pair. The order of the stimulus pairs along the *x* axis corresponds to the order of the stimuli shown in Panel A. Apart from that, format as in Figure 3(a).

A second online survey was conducted to check the robustness and test further predictions of the memory colors effect. In this survey, all six combinations of images were used, as illustrated in Figure 4(a), and 200 observers (age: 30.7 ± 11.5 years; 114 women) participated (see Methods section for details).

Figure 4(b) illustrates the corresponding results. The first two stimulus pairs were the same as in Study 1, and they also reproduced similar results as in Study 1: The relative frequency of choosing the bluish disk (*n–k* = 110) over the gray disk (*k* = 90) was not different from chance in the two-tailed binomial test (*p* = .18). At the same time, the bluish banana (*n–k* = 133) was chosen significantly (*p* = .000004) more often than the grayish banana (*k* = 67). Moreover, McNemar’s exact test showed that the proportion of choosing the blue over the gray banana was significantly different from the proportion of choosing the blue over the gray disk (*p* = .002). The effect size of this McNemar test was very similar to the one observed in the first study (Odds ratio: 3.1; phi = 0.23). In contrast to Study 1, memory color effects could not only be shown for the 114 women (*p* = .01; Odds ratio = 3.2; phi = 0.22), but also for the 86 men (*p* = .04; Odds ratio = 3.0; phi = 0.22). As in Study 1, the difference in memory color effects between women and men was not significant (*z* = −.08; *p* = .94).

These results replicate the results of the first study in support of a memory color effect and against a difference between women and men. The similarity of the results across the two independent studies suggests that the observed evidence for memory color effects is statistically robust. Significant memory color effects for men could only be shown in the second but not in the first study. This might suggest that the sample sizes of male participants (98 in Study 1 and 86 in Study 2) was just too low to show reliable memory color effects. Hence, it seems advisable, as a very rough rule of thumb, that this kind of online test for memory color effects should involve a minimum of 100 observers per group.

The four additional stimulus pairs (Mix 1–4 in Figure 4(a)) allowed for testing further predictions of the memory color effect. In the case of a memory color effect, the gray banana but not the gray disk should appear yellowish, and hence observers should choose the gray disk over the gray banana in the third stimulus pair (Mix 1). Moreover, observers should choose the bluish banana over the bluish disk because the yellow induced by the memory color effect should cancel the banana’s bluishness (Mix 2). Observers should also choose the bluish disk over the yellow banana because the blue bias should make the bluish disk appear grayish while the gray banana should look yellowish (Mix 3). Finally, the comparison between the blue banana and the gray disk (Mix 4) allows for measuring whether the blue banana is perceived differently than the gray disk, which indicates how accurately the amount of blue in the banana cancels the perceived yellowness due to the memory color effect. However, no effects were found for any of the four mixed stimulus pairs (Mix 1–4). In all four cases, both alternatives were chosen almost equally often (i.e., in 50% of responses), and hence, there was no significant difference of the relative frequencies from chance (all *p* > .52).

**Figure 5.**
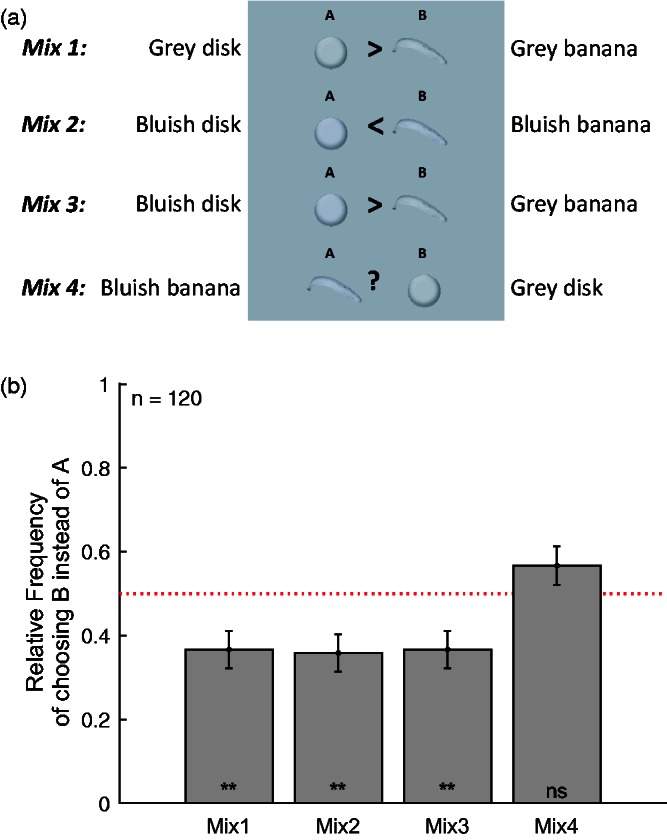
Stimuli and results of supplementary survey of Study 2. Format as in Figure 4.

One may wonder whether this was due to the observers’ understanding the principles of our stimulus design after having seen the two kinds of disks and the two kinds of bananas on the first two pages of the survey. To check this, this survey was reimplemented without the comparison between the two disks and the two bananas (first two stimulus pairs), but only with the four comparisons between disk and banana, that is, Mix 1–4 (Figure 5(a)). A total of 120 observers (age: 30.8 ± 9.1 years; 40 women) took part (see Methods section for details). Results are shown in Figure 5(b). As predicted by the idea that the gray banana looks yellowish, observers chose more often the gray disk (Mix 1) as well as the bluish disk (Mix 3) over the gray banana as shown by the two-tailed binomial test (*k* = 44, and *k* = 43, both *p* = .004). However, the bluish disk was also chosen more often over the bluish banana (Mix 2; *k* = 43, *p* = .002), which contradicts the idea that the bluish banana looks gray due to the memory color effect. Finally, there was no significant difference from chance level for the last stimulus pair (Mix 4; *k* = 68, *p* = .17). Hence, we cannot draw a clear conclusion about the relationship between the gray disk and the bluish banana.

Taken together, the results of this supplementary survey partly confirm (Mix 1 and Mix 3) and partly contradict (Mix 2) the predictions based on the memory color effect. Moreover, these results could be interpreted in another way, namely that overall observers choose the disk over the banana in all comparisons (Mix 1–3, and in tendency also Mix 4). Hence, these results cannot be interpreted unambiguously. They raise the question of whether they are merely a limitation of the present method of using online surveys; or whether they indicate a fundamental feature of memory color effects. It might be worthwhile to further investigate those comparisons on a calibrated monitor, maybe through an adjustment task, in order to clarify this question.

This open question notwithstanding the present two studies revealed reliable memory color effects when observers compared the grayness of a gray banana and a slightly bluish banana (Figure 1). These results replicated the memory color effects found for the banana in the achromatic adjustment task on a calibrated setup (Hansen et al., 2006; Olkkonen et al., 2008).

The present findings should be understood in the context of those found with the achromatic adjustment method (Hansen et al., 2006; Kimura et al., 2013; Olkkonen et al., 2008; Witzel et al., 2011). It might be argued that a simple choice between two alternatives, as in Figures 1 and 2, is more sensitive to judgmental biases. In particular, the method used here did not reveal unambiguous memory color effects when disks were compared with the image of the banana (Mix 1–3). If responses were based on the perceived yellow of the gray banana, those stimulus pairs should also have yielded significant results, but this was not the case. Hence, it is possible that the choices of the blue over the gray banana (Figure 2) were due to the observers’ evaluations and judgements on cognitive rather than perceptual grounds (Firestone & Scholl, 2015). However, such a judgement bias cannot explain why observers adjust the banana to a bluish color in an achromatic adjustment task where the goal of the task (i.e., the gray adjustments) could only be completed through perceptual evaluations and comparisons of the adjusted colors (Witzel et al., in press).

At the same time, the present findings supplement those obtained with the achromatic adjustment method. One could argue that the results in the achromatic adjustment task could potentially be explained by observers having stricter criteria for avoiding typical colors of objects in their adjustments than for avoiding colors in the opponent direction of the typical colors (Zeimbekis, 2013). An asymmetric criterion in evaluating their own color adjustments could result in a shift of the mean adjustment away from the typical color without this being necessarily a perceptual effect. In contrast, the display used here provided a completely gray image of the banana and allowed for direct perceptual comparison between the gray and the bluish banana. In this way, observers did not have to evaluate the accuracy of their responses in the adjustment task. Instead, they could simply choose the completely gray banana over the slightly bluish banana. Nevertheless, they chose the bluish one. Hence, the memory color effect observed in the current study undermines the criticism that memory color effects could be explained by a response bias.

In contrast to the achromatic adjustment method, the present approach offers a fast and easy way to reveal memory color effects. Moreover, the present results show that memory color effects are robust against minor variations in color rendering due to differences across computer monitors. In their latest article, Firestone and Scholl (2015) claimed that the memory color effect does not exist. As a proof, they argued that it cannot be seen in a simple, uncalibrated display with a gray banana shown in the context of yellow bananas. Firestone and Sholl left it to the reader to check the correctness of this statement for themselves with Figure 2(K) of that article. Witzel et al. (in press) have argued on theoretical grounds that there is no hope for revealing a memory color effect with that Figure 2(K). Now, the present studies show empirically that memory color effects can actually be shown with such a simple, uncalibrated display, when done correctly. Everybody, even without any expertise in color rendering and monitor calibration, can take the images in Figures 1 and 2 and verify the existence of memory color effects in a fast and easy online study.

In addition, the simple approach proposed here makes it particularly easy to compare memory color effects across groups of observers. This is particularly true for cross-cultural studies and studies of groups with a low prevalence (e.g., synesthesia, color deficiency, etc.), which would require observers from a large catchment area. With our method, observers can participate online, which avoids traveling of either the experimenter or the participants. This approach is also particularly beneficial for comparing heterogeneous groups, such as women and men, or different professions (artists vs. engineers, etc.). Such comparisons require large samples of participants in order to isolate the feature that characterizes the difference between groups from other individual differences.

More generally, the present studies provide an example for the successful use of online surveys for experimental research on perception. Online studies on perception have advantages and disadvantages when compared with controlled experiments in the laboratory (Woods, Velasco, Levitan, Wan, & Spence, 2015). The present example shows that online surveys provide additional options that supplement and enrich the experimental repertoire.

## Method

### Participants

For the first study, participants were recruited from an email list (CNRS, 2015), and they participated voluntarily. A total of 367 participants completed the survey; 13 of these were excluded because they reported that they have color vision deficiencies or that they do not know whether they have color vision deficiencies. The average age of the remaining 354 observers was 36.8 years (*SD* 12.1 years) and 256 were women.

For the second study, participants were recruited through a commercial online recruitment platform (Isis Software Incubator, 2015) and were paid for participation. Participants were recruited until the sample included 200 observers without known or potential color vision deficiencies. For this, 210 observers completed the survey, 10 of which were excluded to avoid potential color deficiencies. The average age of the 200 observers in the final sample was 30.7 years (*SD* 11.5 years) and 114 were women. 120 additional observers were recruited for the supplementary measurement of the second study (with only stimulus pairs Mix 1–4). They resulted from an original sample of 131 observers, 11 of which were excluded due to known or potential color deficiencies. The average age was 30.8 years (*SD* 9.1 years) and 40 of the 120 observers were women.

### Stimuli

In Study 1, stimuli were the images in Figures 1 and 2. Figures 4(a) and 5(a) show the stimuli in the order of presentation in Study 2. Since display size should be large enough, participants were instructed to use only desktops, laptops, or tablets, but not mobile phones.

### Procedure

Observers were asked the following: “Which disk looks gray?” The instructions read: “See image below. This question assesses the exact perception of reference gray. One of these two disks will look more purely gray, and the other may appear to have some other color mixed in. If none of the disks looks perfectly gray to you, please choose the most gray one.” The observers answered by ticking either A or B. The same text and answer options were presented for the banana (the term *disk* was replaced with *banana*). The questions were part of a larger online survey with other questions on color perception. The survey started with questions about gender, age, and color deficiency. In Study 1, the question about the disk was presented at the end of the first page. The banana was presented at the end of a third page, with other questions (not reported here) in between. In Study 2, all six stimuli were shown on different pages of the survey. In this way, images and answers for the disks and bananas could not be compared directly.

## Figures and Tables

**Table:** 

	Gray disk	Blue disk
Gray banana	a	b
Blue banana	c	d

## Author’s note

The author is currently affiliated to Allgemeine Psychologie, Justus-Liebig Universität, Gießen, Germany.

## Appendix: Explanation of Extended McNemar Test

Contingency table for illustration:

According to McNemar’s approach (McNemar, 1947), the null hypothesis is:

b/(b+c)=c/(b+c),henceb/(b+c)=0.5

This can be tested through a binomial distribution or, for large samples, with a χ^2^ distribution with 1 degree of freedom. The latter is equivalent to a normal distribution used in *z* tests. Here, we compare a difference in repeated measurements (disk vs. banana) across groups. Hence, the null hypothesis is:

b1/(b1+c1)=b2/(b2+c2)

where b1 and c1 are the response frequencies of the first group (e.g., women) and b2 and c2 those of the second group (e.g., men). Since b1/(b1+c1) and b2/(b2+c2) are normally distributed, the test of this null hypothesis comes back to a *z* test between two proportions. To accomplish those *z* tests, the pooled standard deviation of the samples, that is, the pooled standard error was calculated:

$$\text{SEM}=\sqrt{\frac{\text{p}1*(1-\text{p}1)}{\text{n}1}+\frac{\text{p}2*(1-\text{p}2)}{\text{n}2}}$$

where n1 = b1 + c1; p1 = b1/(b1 + c1); n2 = b2 + c2; and p2 = b2/(b2 + c2).

Then, z is simply:

z=(p1-p2)/SEM

The *p* value is obtained from the cumulative normal distribution (multiplied by 2 for two-tailed tests):

p=2*(1-normcdf(abs(z),0,1));

The complete Matlab code for this extended McNemar test is:

function ztab  =  McNemar_extender(b1, c1, b2, c2)

% 2016.06.13 * [cw]

p1 = b1  /  (b1 + c1);

p2 = b2  /  (b2 + c2);

n1 = b1 + c1;

n2 = b2 + c2;

ztab  =   ztester2 (p1, n1, p2, n2);

function ztab  =  ztester2(p1, n1, p2, n2)

% 2016.06.13 * [cw]

% pooled SD of sampling (i.e., sem):

SD = sqrt(p1*(1−p1) /n1+p2*(1−p2)/n2);

z = (p1−p2)/SD;

p = 2 * (1-normcdf(abs(z), 0, 1)); % two-tailed

ztab = dataset(z, p);
