# ERRATUM to An Easy Way to Show Memory Color Effects

**DOI:** 10.1177/2041669516688509

**Published:** 2017-01-01

Owing to errors made by SAGE, the following article contains errors.

Christoph Witzel (September-October 2016). An Easy Way to Show Memory Color Effects.

i-Perception, 7(5), 1–11. (DOI: 10.1177/2041669516663751).

SAGE apologises to the author and to the readers. The following corrections apply:

Figures 1 and 2 are now correctly displayed as:

**Figure 1.**
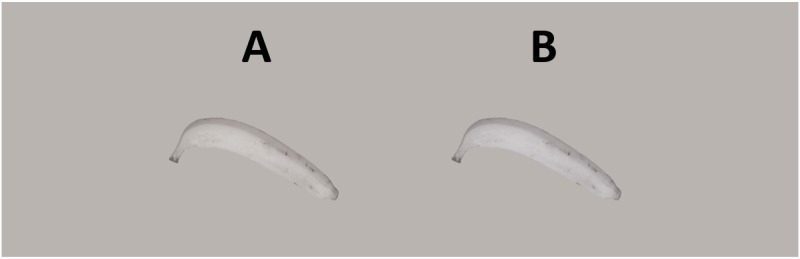
Main stimulus display. The left banana is completely gray (like the background), the right one slightly bluish. Which banana looks gray? If neither looks gray choose the most gray one.

**Figure 2.**
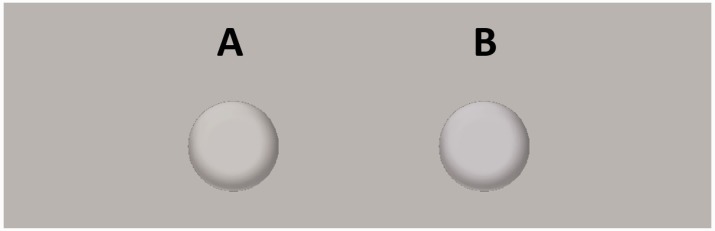
Control stimuli. Chromatic properties as for stimuli in Figure 1.

These corrections will be included in all subsequent versions of the article online.

